# Account of the Four-Legged Child

**Published:** 1868

**Authors:** J. Myrtke Corban


*Account of the Four-Legged Child*, J. Myrtle Corban.

NASHVILLE, TENN., June 16, 1868.

The undersigned, in response to the request of a number of physicians and the relatives and friends of the unfortunate subject of this investigation, give the following testimony: The infant, J Myrtle Corban, has four legs and two distinct female organs of generation, with two external openings of the double rectum. The external genito-urinary organs are as distinct as if they belonged to two separate human beings. The fæces and urine are passed (most generally simultaneously, particularly the urine,) from both external urinary and intestinal openings, situated respectively between the left and right pairs of legs.

The head and trunk are those of a living, well-developed, healthy, active infant of about five weeks, whilst the lower portion of the body is divided into the members of two distinct individuals, near the junction of the spinal column with the *os sacrum.* As far as our examination could be prosecuted in the living child, we are led to the belief that the lower portion of the spinal column is divided or cleft and there are *two pelvic arches supporting the four limbs*, which are situated upon the same plane.

Photographs of this infant have been made by the advice and under the supervision of one of our number.

The reality in this case surpasses expectation, and we are of the opinion that this interesting *living monstrosity* exceeds in its curious manifestation of the powers of nature in abnormal productions, the celebrated "Siamese Twins."

JOSEPH JONES, M. D.
Prof. of Phys. and Path., U. of Nashville.
PAUL F. EVE, M. D.
Prof. of Surg., University of Nashville.

*Further remarks by Professors Jones and Eve, for this Journal:*—Josephino Myrtle is the third offspring of W. H. and Nancy Corban, aged twenty-five and thirty-four, the wife being the senior by nine years. They are so much alike in appearance, having red hair, blue eyes, and very fair complexion, as to produce the impression of their being blood kin, which, however, is not the case. Mrs. Corban is from North Alabama, had borne one child to a former husband, the child having dark coloring, and resembling mostly the father, who had black hair and eyes. Her three children are all girls; the one already alluded to, now six years old, another three, and this *infant monstrosity*, now to be more minutely described, born the 12th of May, 1868, in Lincoln County, Tennessee, five weeks ago.

Mr. Corban is a Georgian, served in the Confederate army through the war, and was severely wounded in the right arm and left hand. The parents are in fair health, though the mother is *anæmic.* She recollects no fright or disturbance during her pregnancy. The presentation was fortunately the head, which accounts for the preservation of the life of the child. It would be curious to speculate on the trouble which might have been produced had the feet or breach presented, while the result, in all probability, would have proved fatal to the infant, and possibly to the mother. Mrs. Corban says that there was nothing peculiar in the labor or delivery. When three weeks old, the child weighed ten pounds. It now nurses healthily, is thriving well, and we saw it urinate simultaneously, between the *two pairs of labia of the two vaginæ*, situated about six inches apart. From the crown of the head to the *umbilicus* the child measures twelve inches, and from this point to the toes of the right and left external feet, eleven inches. From the *umbilicus* up, all is natural and well formed; all below this, extraordinary and unnatural. An inch below the navel is a mark of apparent failure for a second one. There are four distinct well developed, lower extremities. They exist in pairs on both sides of the median line which resembles the cleft of an ordinary pair of legs; but here there are no marks whatever of *anus* or *genital* organs, and upon pressure we discover no *os coccygis* or *sacrum.* The outer legs of both sides are the most natural of the four, (though the foot of the right one is clubbed,) but are widely separated by the two supernumerary ones, which are less developed, except at their junction with the body, from which they taper to the feet and toes more diminutive and which are turned inwards. One toe is bifid on the left extra inward extremity. At birth these extra legs were folded flat upon the abdomen. We are led to believe that there are *two uteri as well as two recti;* in fact that the pelvic organs are double. Of course a minute dissection would alone expose the true condition of these parts.

Should this infant reach maturity and the internal generative organs be double, there is nothing to prevent conception on both sides. The first difficulty will, however, be in her walking. The outer, or external legs, may be used for progression; the inner or inturned ones, probably never. These might be successfully amputated at the knees, or higher up.

One of us recollects of being in London, in January, 1830, at an exhibition of the Siamese Twins, when Sir Astely Cooper gave an opinion adverse to an operation with a view to separate them, but which has always appeared to us feasible and without much risk of *peritonitis;* an operation, too, which should undoubtedly be performed in case of the death of one of them for no medical man believes in the vulgar impression that they must die simultaneously. In the present case all surgical interferences is, of course, out of the question, except that alluded to—removal of the extra legs.

Cases somewhat similar to the above have occured and been described. Rokitansky refers to two completely distinct bodies conjoined at their *ossa sacra* or *coccyges*, as in the well known Hungarian Sisters, Helena and Judith, born in 1701, who survived their twenty-second year.

Geoffrey St. Hilaire, alludes to cases of a trunk with two heads, some even Janus-like, having four upper and four lower extremities.

The case, however, recalled most vividly by Josephine Myrtle, is that of Rita-Christina, well known in Europe, and accurately described in this country years ago by Prof. Meigs. In this wonderful instance, there were two heads, two necks, four arms, but only two legs; and was thus the reverse of our case. From the *umbilicus* down, there was one well formed child, but above this all the organs were double; in reality there existed two beings. The *rectum* and bladder were common to both, but all else in the trunk was double and distinct. One would sleep while the other played, etc., for they had *two spinal marrows*, *two brains*, *two hearts*, but the last two occupied a common *pericardium*. Unfortunately, after surviving a little over a year, one sickened and died, when the other, then in health, instantly expired.

Rita and Christina were born in Sardina, 1829, and described by Dr. De Michaelis, Professor of Surgery in the Royal University of Sassari, and lived eighteen months.

The late Prof. J. C. Warren, of Boston, first described the Siamese twin brothers, when purchased of their mother by Capt. Coffin and Mr. Hunter, (joint owners) and brought to that city, in 1829.—*Rich. and Louisvillé Med. Journal.*

[In the Museum of the Atlanta Medical College, can be seen, a well developed female child, having two distinct and well formed heads and necks. The two necks seem to unite at the upper portion of the dorsal spine; and the child has no other deformity in any respect.

The history of this very remarkable *monster* is not known by any one connected with the College. When deposited in the Museum, by Dr. J. Gilbert, of this city, some ten years since, the thoracic and abdominal cavities had been opened, and the child preserved perfectly in alcohol. No positive evidences of its having breathed, exist, but from appearances of the body it would not be unreasonable to suppose that it had lived several days after birth. Dr. G. obtained this body from persons professing to know nothing of its mother; and from their account of the circumstances under which it was found, in all probability the parent had deserted it in order to conceal the shame of illegitimacy.–Eds.]

